# Contrast-enhanced CT radiomics for predicting lymph node metastasis in pancreatic ductal adenocarcinoma: a pilot study

**DOI:** 10.1186/s40644-020-0288-3

**Published:** 2020-01-30

**Authors:** Ke Li, Qiandong Yao, Jingjing Xiao, Meng Li, Jiali Yang, Wenjing Hou, Mingshan Du, Kang Chen, Yuan Qu, Lian Li, Jing Li, Xianqi Wang, Haoran Luo, Jia Yang, Zhuoli Zhang, Wei Chen

**Affiliations:** 1 Department of Radiology, Southwest Hospital, Army Medical University, Chongqing, 400038 China; 2 Department of Radiology, Sichuan Science City Hospital, Mianyang, Sichuan China; 3 Department of Medical Engineering, Xinqiao Hospital, Army Medical University, Chongqing, China; 4 Hepatopancreatobiliary Surgery, Southwest Hospital, Army Medical University, Chongqing, China; 5 Department of Radiology, Feinberg School of Medicine, Northwestern University, Chicago, IL USA

**Keywords:** Pancreatic ductal adenocarcinoma, Radiomics, CT, Lymph node metastasis

## Abstract

**Background:**

We developed a computational model integrating clinical data and imaging features extracted from contrast-enhanced computed tomography (CECT) images, to predict lymph node (LN) metastasis in patients with pancreatic ductal adenocarcinoma (PDAC).

**Methods:**

This retrospective study included 159 patients with PDAC (118 in the primary cohort and 41 in the validation cohort) who underwent preoperative contrast-enhanced computed tomography examination between 2012 and 2015. All patients underwent surgery and lymph node status was determined. A total of 2041 radiomics features were extracted from venous phase images in the primary cohort, and optimal features were extracted to construct a radiomics signature. A combined prediction model was built by incorporating the radiomics signature and clinical characteristics selected by using multivariable logistic regression. Clinical prediction models were generated and used to evaluate both cohorts.

**Results:**

Fifteen features were selected for constructing the radiomics signature based on the primary cohort. The combined prediction model for identifying preoperative lymph node metastasis reached a better discrimination power than the clinical prediction model, with an area under the curve of 0.944 vs. 0.666 in the primary cohort, and 0.912 vs. 0.713 in the validation cohort.

**Conclusions:**

This pilot study demonstrated that a noninvasive radiomics signature extracted from contrast-enhanced computed tomography imaging can be conveniently used for preoperative prediction of lymph node metastasis in patients with PDAC.

## Background

Pancreatic ductal adenocarcinoma (PDAC) is an aggressive disease and the fourth leading cause of cancer-related death worldwide, although it is predicted to become the second leading cause by 2030 [1, 2]. PDAC has a poor prognosis, and the 5-year survival rate for all stages is approximately 6%; whereas after surgical resection, the 5-year survival rate can reach 25% [3–6]. Lymph node (LN) metastasis is an independent prognostic factor in PDAC, and preoperative chemotherapy can improve the prognosis of node-positive patients [7–10]. Therefore, accurate preoperative identification of LN involvement in patients with PDAC is crucial for predicting prognosis and for designing better treatment strategies. However, postoperative pathological specimens are generally needed for detecting LN metastasis. In recent years, novel serum markers such as MMP7, MUC1, MUC2, and NLR have been proposed for detecting LN metastases preoperatively in PDAC patients [11–13]. However, their clinical application is limited because of technical and accuracy issues.

Computed tomography (CT), which is commonly used in preoperative work-up, is important for the preoperative diagnosis of LN metastasis in PDAC patients, in clinical practice. CT relies on the identification of enlarged LNs to diagnose metastasis. However, the significance of enlarged LNs in PDAC is not well defined. Enlarged LNs can be due to local inflammation or biliary obstruction, and metastatic LNs may not be enlarged [14–17]. Radiomics is a rapidly developing discipline that converts medical images into high-dimensional, mineable data via high-throughput extraction of quantitative features to reflect cellular and biological changes in tissues [18–21]. Two previous studies showed that CT radiomics can predict the malignant potential of intraductal papillary mucinous neoplasms, with important implications for clinical decision-making [22, 23]. However, standardized studies, including large sample sizes are needed to confirm the reliability of this method.

The purpose of this study was to investigate the value of radiomics features extracted from contrast-enhanced CT (CECT), combined with clinical information, for the preoperative prediction of LN metastasis in patients with PDAC.

## Materials and methods

### Patients

**Fig. 1:**
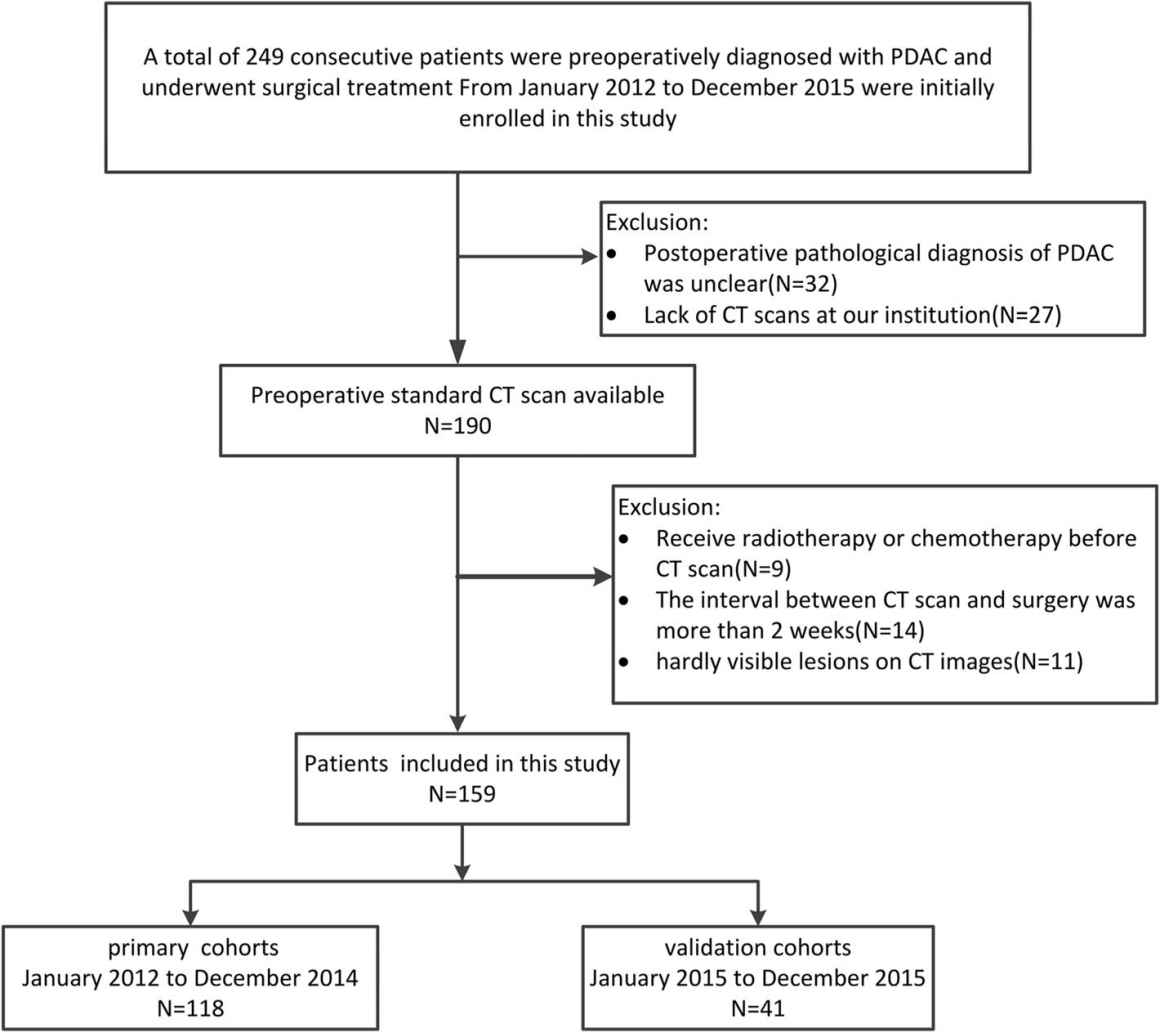
Flow chart of the study population, with exclusion criteria

This retrospective study included a primary cohort of patients who underwent surgical resection of PDAC, between January 2012 and December 2014 at Southwest Hospital (Chongqing, China). A patient recruitment flowchart and inclusion and exclusion criteria are described (Fig. 1)*.* The primary cohort comprised 118 patients, including 82 men and 36 women with a mean age of 57.75 ± 10.28 years. Between January 2015 and December 2015, 41 consecutive patients were recruited using the same criteria as that used for the primary cohort; they constituted the independent validation cohort, comprising 23 men and 18 women with a mean age of 58.32 ± 9.85 years. Ethical approval was obtained from the Ethics Committee of Southwest Hospital, Third Military Medical University (approval No.KY201802) and informed consent requirements were waived.

### Clinical and histopathological analyses

Surgical specimens were evaluated for pathological grading and LN metastasis according to the World Health Organization 2010 and AJCC 8th edition criteria [24, 25]. Clinical data included age, gender, carcinoembryonic antigen (CEA) levels, cancer antigen-19-9 (CA19–9) levels, and total bilirubin (TBIL) levels. The threshold values were 5 μg/L for CEA, 35 U/mL for CA 19–9, and 22 μmol/L for TBIL, based on normal ranges as determined in our hospital.

### CT protocols and radiographic evaluations

Patients with PDAC were scanned on a dual-source MDCT scanner (FLASH, Siemens Healthineers). The scanning protocol was as follows: 120 kVp, 300 mA, 0.6–0.8 pitch and 128 × 0.6 mm. CT scans of patients included both arterial and venous phases according to institutional protocols. Patients received an injection of 100–120 mL of iohexol (Omnipaque, GE Healthcare) via the cubital vein before scanning. Arterial phase imaging was performed using bolus triggering, approximately 30–40 s after injection, and venous phase imaging was performed approximately 60–70 s after injection. Images were reconstructed into 2 mm sizes for radiographic evaluation and reconstructed into 1 mm sizes for segmentation and radiomics analysis.

CECT images were retrospectively analyzed by two radiologists (one with 8 years of abdominal imaging experience and one with 10 years of abdominal imaging experience) who were blinded to the clinical and pathological data. Assessments included the following: (1) lesion location (head, body or tail); (2) LN status based on abdominal imaging evaluation criteria (location, size, shape, and LN enhancement) [26, 27]. If the evaluation results were different, the final data were obtained after consultation between the two observers. The original evaluation results were retained for consistency analysis.

### Image segmentation and radiomics feature extraction

**Fig. 2:**
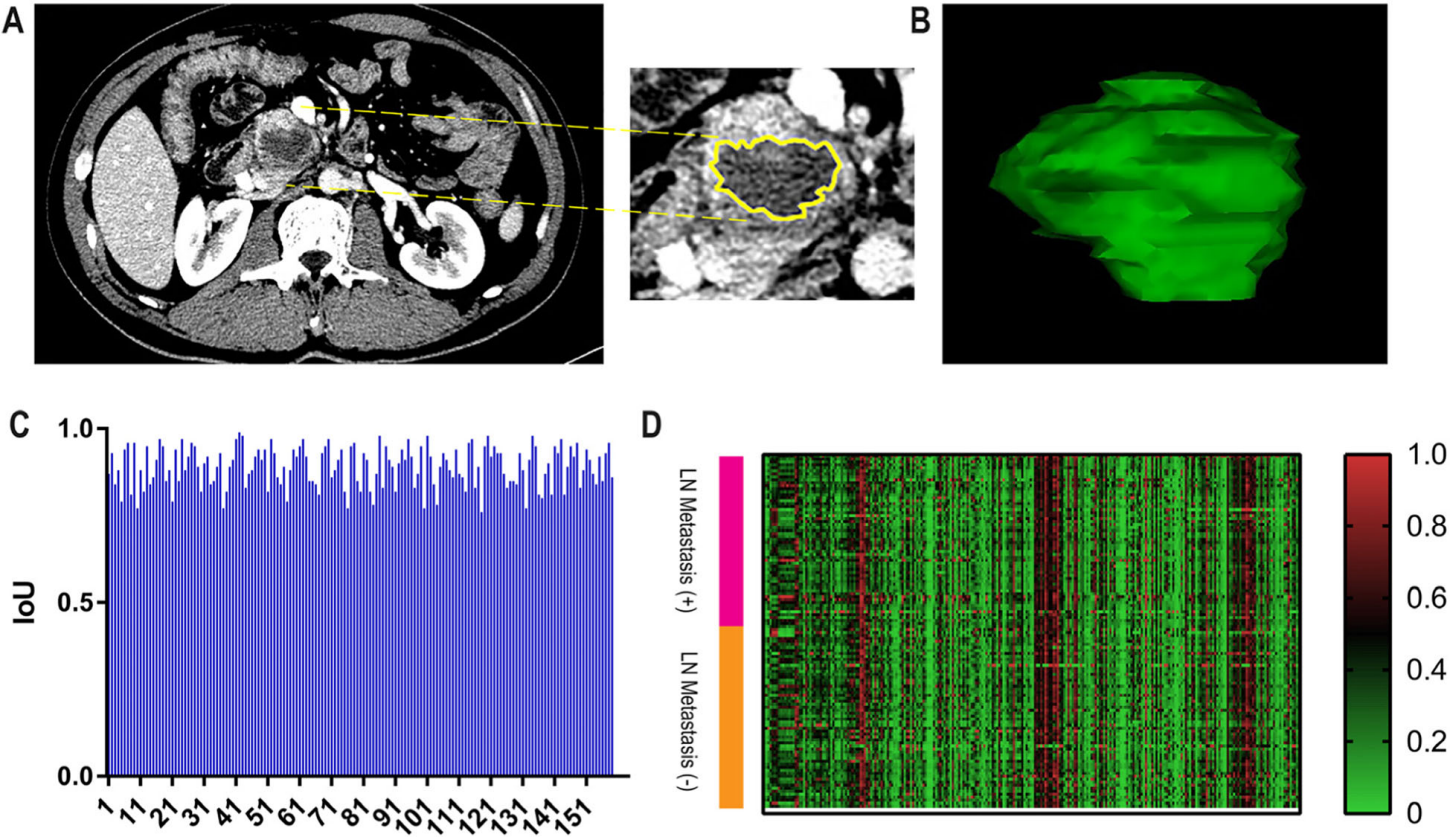
Tumor segmentation on CT images with pancreatic ductal adenocarcinoma and heat map. **a** Segmentation on axial image slice-by-slice (yellow regions). **b** Three-dimensional view of the tumor. **c** IoU scores of each patient. **d** Heat map representation of radiomics features on the x-axis and cases on the y-axis. Right color bar represents color coding of Z-scores of each radiomics feature on 118 cases, in the primary cohort

The venous phase image was selected for image segmentation because it was more accurate for displaying the lesion boundary. The region of interest (ROI) of the lesions was delineated manually by two radiologists using in-house developed computer-aided segmentation tools (QJImageEditor, Quanjing Medical Co. Ltd.) and segmented in 3D. The ROI included cystic and necrotic lesions, whereas blood vessels and lymph nodes were not included (Fig. 2). Subsequent feature extraction was performed to select the segmentation area common to both radiologists. The original segmentation results were retained for consistency analysis.

The pyradiomics package (http://www.radiomics.io/pyradiomics.html) was used for feature extraction.

### Statistical analysis

Statistical analysis was performed using R software (version 3.5.3, http://www.r-project.org). Significance was two-sided, and *p* <  0.05 was considered statistically significant.

### Consistency test

The Kappa consistency test examined the diagnostic results of two radiologists regarding lesion location and LN status. To evaluate the consistency of segmentation results, intersection-over-union (IoU) was used as an evaluation metric and calculated with the following formula:

$$ \mathbf{IoU}\left({\mathbf{a}}_{\mathbf{A}},{\mathbf{a}}_{\mathbf{B}}\right)=\frac{{\mathbf{a}}_{\mathbf{A}}\mathbf{\cap}{\mathbf{a}}_{\mathbf{B}}}{{\mathbf{a}}_{\mathbf{A}}\cup {\mathbf{a}}_{\mathbf{B}}} $$

where a_A_ and a_B_ represent the segmented areas of the same patient’s data obtained by each radiologist.

### Feature selection and radiomics signature construction

The Z-score was standardized for the extracted features. The least absolute shrinkage and selection operator (LASSO) method, which is suitable for the reduction of high dimensional data [28], was used to select optimal predictive features from the primary cohort. Its performance was verified using a 10-fold cross-validation approach. Features with nonzero coefficients in the LASSO regression model were selected. Then, multivariable logistic regression analysis was used to build a prediction model base on the feature selected. Receiver operating characteristic (ROC) curves and area under the curve (AUC) were used to evaluate the predictive ability of the model, and its verification on the validation cohort. The radiomics signature of each patient was the linear combination of selected features weighted by their coefficients, denoted as:

$$ \sum \limits_{\boldsymbol{i}=\mathbf{1}}^{\mathbf{n}}{\boldsymbol{\beta}}_{\mathbf{0}}+{\boldsymbol{\beta}}_{\boldsymbol{i}}\times {\mathbf{X}}_{\boldsymbol{i}} $$

Where *β*_0_ is the intercept, *X*_*i*_ is the *i* th selected feature and *β*_*i*_ is the coefcient of the *i* th selected feature.

### Establishment of clinical and combined prediction models

Univariate analysis assessed the relationship between the clinical characteristics of the patients and LN metastasis in the primary cohort, including age, gender, pathological grading, CEA levels, CA19–9 levels, TBIL levels, CT-reported lesion location and CT-reported LN status. Continuous variables were assessed using independent t-tests or Mann-Whitney U tests, and categorical variables were evaluated using chi-square tests, Kruskal-Wallis tests, or Fisher’s exact tests. Statistically significant variables were included in the multivariate logistic regression analysis, and clinical predictive models were established. The combined prediction model was built by integrating the radiomics signature and the selected clinical characteristics.

### Model validation and evaluation

The predictive ability of the clinical and combined prediction models was assessed in the primary cohort using ROC curve analysis. Integrated discrimination improvement (IDI) was performed to determine whether differences in predictive ability between the two models were statistically significant. The best performing model was then presented as a nomogram. The logistic regression formula used in the primary cohort was applied to the validation cohort for verification.

A calibration curve was plotted to assess consistency between the estimated probability and the actual rate of LN metastasis, together with a Hosmer-Lemeshow test in the two cohorts [29]. A decision curve analysis was performed to evaluate the clinical usefulness of the nomogram, by quantifying the net benefits at different threshold probabilities [30].

## Results

### Consistency test results

Both radiologists showed good consistency in determining lesion locations and LN status (kappa coefficient = 0.914 and 0.897, respectively). The IoU scores of each patient are shown (Fig. 2). The average IoU score was 0.89, indicating that consistency was high.

### Clinical characteristics

There were no significant differences in the rate of LN metastasis (44.07 and 41.46% in the primary and validation cohorts, respectively, *p* = 0.772) and clinical characteristics between the two cohorts, which confirmed their use as primary and validation cohorts. The differences in clinical characteristics between the LN metastasis-positive group and LN metastasis-negative group were not significant, except for CT-reported LN status and pathological grades in the primary and validation cohorts (Table 1).

**Table 1:** Clinical characteristics of patients in the primary cohort and validation cohort

Characteristic	Primary Cohort		p	Validation Cohort		p
	LN Metastasis (+)	LN Metastasis (−)		LN Metastasis (+)	LN Metastasis (−)	
Age, mean ± SD, years	58.75 ± 10.07	56.95 ± 10.44	0.348	59.12 ± 9.69	57.75 ± 10.13	0.667
Gender, No. (%)			0.647			0.767
Male	35 (67.3)	47 (71.2)		10 (58.8)	13 (54.2)	
Female	17 (33.7)	19 (28.8)		7 (41.2)	11 (45.8)	
CEA level, No (%)			0.185			0.273
Normal	41 (78.8)	58 (87.9)		10 (58.8)	18 (75.0)	
Abnormal	11 (21.2)	8 (12.1)		7 (41.2)	6 (25.0)	
CA19–9 level, No (%)			0.116			0.529
Normal	13 (25.0)	9 (13.6)		5 (29.4)	5 (20.8)	
Abnormal	39 (75.0)	57 (86.4)		12 (70.6)	19 (79.2)	
TBIL level, No (%)			0.281			0.729
Normal	19 (36.5)	18 (27.3)		4 (23.5)	8 (33.3)	
Abnormal	33 (63.5)	48 (72.7)		13 (76.5)	16 (66.7)	
Lesion location, No (%)			0.595			0.262
Head	43 (82.7)	52 (78.8)		15 (88.2)	17 (70.8)	
Body or tail	9 (17.3)	14 (21.2)		2 (11.8)	7 (29.2)	
CT-reported LN status, No (%)			0.020*			0.019*
LN- positive	34 (65.4)	29 (43.9)		12 (70.6)	8 (33.3)	
LN- negative	18 (34.6)	37 (56.1)		5 (29.4)	16 (66.7)	
Pathological grade			0.008*			0.022*
Well	8 (15.4)	22 (33.3)		3 (17.6)	11 (45.8)	
Moderately	17 (32.7)	24 (36.4)		6 (35.3)	9 (37.5)	
Poorly	27 (51.9)	20 (30.3)		8 (47.1)	4 (16.7)	

Abbreviations: *CEA* carcinoembryonic antigen, *CA19–9* cancer antigen-19-9, *TBIL* total bilirubin, *CT* computed tomography, *LN* lymph node, *SD* standard deviation

* highlights the *p* values that are smaller than 0.05

### Feature selection and radiomic signature construction

**Fig. 3:**
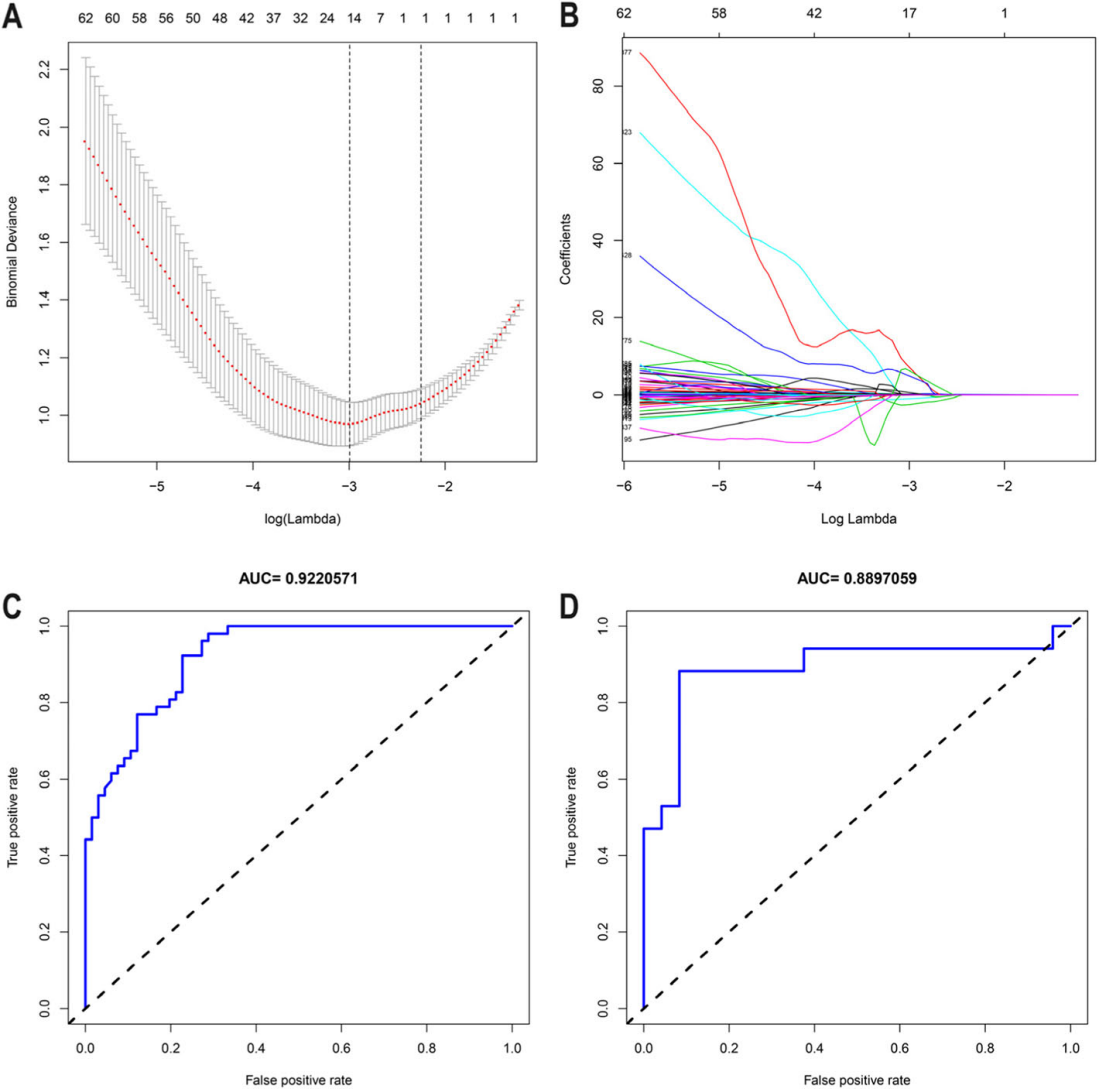
Radiomics feature selection using the least absolute shrinkage and selection operator (LASSO) binary logistic regression model. **a** Optimal parameter (lambda) selection in the LASSO model used 10-fold cross-validation via minimum criteria. The partial likelihood deviance (binomial deviance) curve was plotted versus log (lambda). Dotted vertical lines were drawn at the optimal values using the minimum criteria and the 1 SE of the minimum criteria (the 1-SE criteria). **b** LASSO coefficient profiles of the 2041 features. A coefficient profile plot was produced against the log (lambda) sequence. A vertical line was drawn at the value selected, using 10-fold cross-validation, where optimal lambda resulted in 15 features with nonzero coefficients. **c** ROC curves of radiomics signatures in primary cohorts. **d** Validation cohort

The results of the 2041 radiomics features for both LN metastasis-positive and -negative cases in the primary cohort are shown (Fig. 2). The heat map represents a color-coded array of all feature values (x-axis) in all cases (y-axis). Because there were redundant feature groups that may have affected the classification, we used the LASSO method and selected 15 optimal features with nonzero coefficients in the primary cohort, for constructing the radiomics signature prediction model (Fig. 3). The coefficients of each feature of the model are shown (Table 2). In the ROC analysis, the radiomics signature prediction model yielded an AUC of 0.922 [95% confidence interval (CI), 0.878–0.967] in the primary cohort and 0.890 (95% CI, 0.769–1) in the validation cohort (Fig. 3).

**Table 2:** List of selected feature parameters for establishing the radiomics signature

Feature name and intercept	Coefficient
Intercept	−0.353
original_firstorder_Skewness	−0.833
log-sigma-1-0-mm-3D_glszm_LowGrayLevelZoneEmphasis	0.404
log-sigma-1-0-mm-3D_ngtdm_Busyness	0.379
wavelet-LLL_glcm_JointAverage	−14.890
wavelet-LHL_glszm_SmallAreaLowGrayLevelEmphasis	−0.127
wavelet-LHH_firstorder_Skewness	1.068
wavelet-LHH_glcm_Imc1	9.466
wavelet-LHH_glcm_Imc2	2.352
wavelet-LHH_glszm_SmallAreaEmphasis	−0.462
wavelet-HLL_firstorder_Maximum	−4.677
exponential_firstorder_Energy	−0.937
exponential_glszm_SizeZoneNonUniformity	3.390
gradient_glcm_Idn	2.041
gradient_glszm_SmallAreaLowGrayLevelEmphasis	0.091
lbp-3D-k_glszm_SmallAreaLowGrayLevelEmphasis	3.205

### Establishment, validation and evaluation of clinical and combined prediction models

**Fig. 4:**
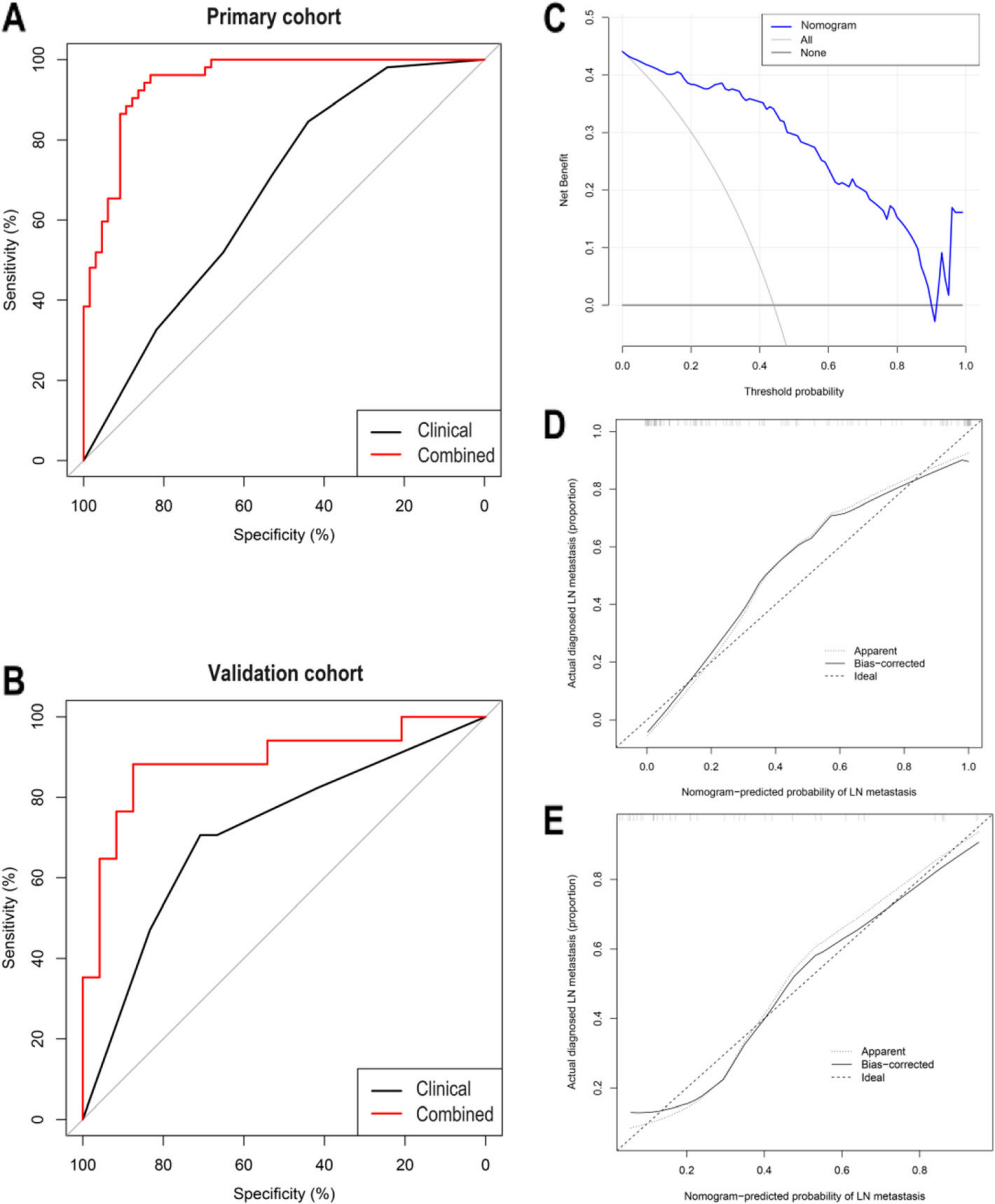
ROC curves of clinical and combined prediction models in both cohorts; decision curve analysis for the combined prediction model in the primary cohort, and calibration curve analysis for the combined prediction model in both cohorts. **a** ROC curves of clinical and combined prediction models in the primary cohort. **b** ROC curves of clinical and combined prediction models in the validation cohort. **c** Decision curve analysis for the nomogram. Nomogram for the combined prediction model in the primary cohort. To use this nomogram, first locate the CT reported LN status, then draw a line straight up to the points axis on the top to get the score associated with negative or positive. Repeat the process for the other covariates (pathological grade and radiomic signatures). Add the score of each covariate together and locate the total score on the total points axis. Next, draw a line straight down to the “probability of LN metastasis” axis at the bottom to obtain the probability. The y-axis measures the net benefit. The blue line represents the nomogram. The gray line represents the assumption that all patients have LN metastases. The thin black line represents the assumption that no patients have LN metastases. The decision curve showed that if the threshold probability of a patient and a doctor is 1 and 89%, respectively, using this nomogram to predict LN metastasis risk adds more benefit than the intervention-all-patients scheme or the intervention-none scheme. **d** Calibration curve analysis for the combined prediction model in the primary cohort and **e** validation cohort. The x-axis represents the predicted LN metastasis risk. The y-axis represents the actual diagnosed LN metastases. The diagonal dotted line represents a perfect prediction by an ideal model. The solid line represents the performance of the combined prediction model, of which a closer fit to the diagonal dotted line represents a better prediction

The radiomics signature, CT-reported LN status, and pathological grades were subjected to multivariable logistic regression analysis in the primary cohort (Table 3). Then, in the two cohorts, the clinical prediction model was built based on the two clinical characteristics, and the combined prediction model was built, based on the two clinical characteristics and radiomics signature. The clinical prediction model yielded an AUC of 0.666 (95% CI, 0.569–0.762) in the primary cohort and 0.713 (95% CI, 0.548–0.878) in the validation cohort. The combined prediction model yielded an AUC of 0.944 (95% CI, 0.905–0.982) in the primary cohort and 0.912 (95% CI, 0.778–1) in the validation cohort (Fig. 4). The IDI value of 0.5046 (95% CI, 0.4106–0.5986, *p* <  0.0001) in the primary cohort and 0.3294 (95% CI, 0.1714–0.4875, *p* < 0.0001) in the validation cohort indicated a significantly improved predictive ability of the combined prediction model, when compared with the clinical prediction model.

**Table 3:** Multivariable logistic regression analyses

Intercept and variable	Combined prediction model in the primary cohort		
	Coefficient	Odds ratio (95% CI)	p
Intercept	−0.461	–	0.499
Radiomic signature	3.533	34.233 (7.344~159.575)	< 0.001
CT-reported LN status	1.130	3.095 (0.941~10.174)	0.063
Pathological grade	0.473	1.605 (0.755~3.412)	0.219

**Fig. 5:**
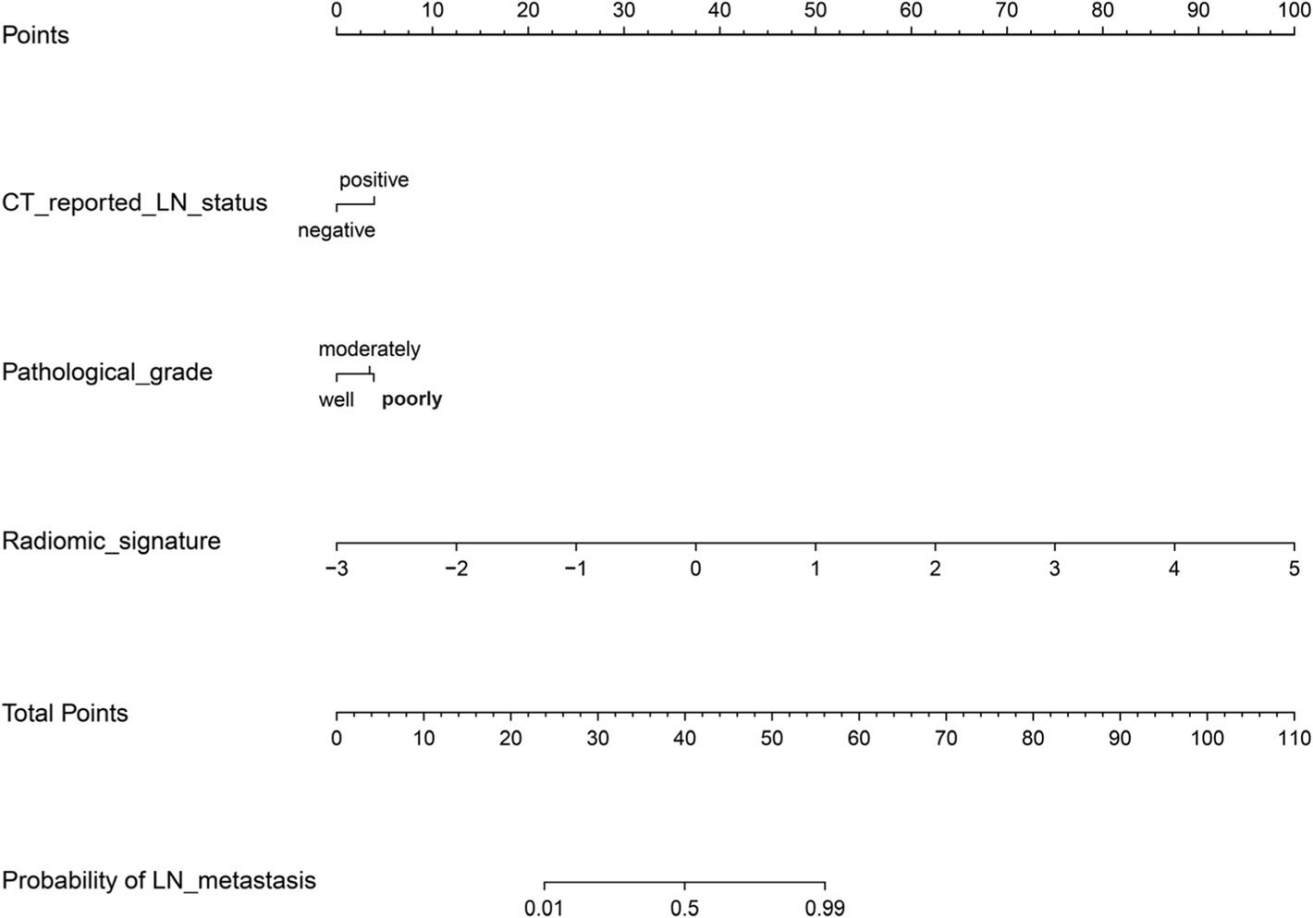
Nomogram for the combined prediction model in the primary cohort

A nomogram was built based on the combined prediction model in the primary cohort (Fig. 5). The decision curve analysis for the nomogram showed that if the threshold probability of a patient and a doctor is 1 and 89%, respectively, then by using the radiomics nomogram to predict LN metastases, this adds more benefit than either the treat-all-patients scheme or the treat-none scheme. Within this range, the net benefit was comparable with several overlaps on the basis of the nomogram. The calibration curve of the combined prediction model in the two cohorts demonstrated good agreement between prediction and observation. The Hosmer-Lemeshow test yielded a non-significant statistic (*p* = 0.215 and 0.462, respectively) (Fig. 4).

## Discussion

This study determined the correlation between certain radiomic signatures and preoperative LN metastasis, in a retrospective analysis of 159 patients with PDAC. A combined prediction model, based on the preoperative CECT imaging radiomics signature, CT-reported LN status, and pathological grade, was built to identify patients with LN metastasis before surgery. AUC values of 0.944 in the primary cohort and 0.912 in the validation cohort were obtained. This suggested that this model can be of clinical value for the diagnosis of preoperative LN metastasis in patients with PDAC.

The TNM-based staging system of the American Joint Committee on Cancer is commonly used for PDAC staging, with LN status being an important component [31]. Preoperative lymph node metastasis is an independent prognostic factor for PDAC and has an important impact on the choice of treatment strategies [7, 8, 32–35]. A French prospective multicenter study that included 147 patients, indicated that preoperative LN involvement had a greater effect on prognosis than resection margin status [36]. Another study showed that the prognosis of patients with preoperative LN metastasis positive PDAC can be improved by neo-adjuvant therapy for fibrosis of LNs [35]. Therefore, accurate preoperative diagnosis of LN status in patients with PDAC has important clinical significance. CA 19–9 levels are independent predictive indicators for LN metastasis [37], however, in this study, CA 19–9 levels had no statistical significance (*p* > 0.05). This observation may be related to sample size, therefore large-scale clinical trials are needed to verify this result. Traditionally, the diagnosis of preoperative LN status in PDAC depends on imaging examinations. Studies have shown that the accuracy of CECT diagnosis for LN metastasis is 48%, and even with 18-fluorodeoxyglucose PET/CT, the accuracy is only approximately 68% [38]. In this study, the diagnostic accuracy of CT-reported LN status in the primary cohort was 60, and 68% in the validation cohort. The AUCs of the clinical prediction model based on CT-reported LN status, and pathological grade in the primary cohort and validation cohort were 0.666 and 0.713, respectively. Considering that pathological grade is obtained from postoperative specimens, its predictive ability will be further reduced after removal pathological grade.

Radiomics has been recognized as an important technology for the conversion of digital medical images to mineable high-dimensional data, and great achievements have been made in recent years [19, 20, 39]. The application of radiomics to PDAC has generated optimism, but it is also challenging because of nonspecific clinical presentation and subtle imaging findings. Previous studies on the application of radiomics to PDAC have focused on prognostic assessments and differential diagnosis [40–43]. The current study used radiomics features of the entire 3D volume to assess preoperative LN status in patients with PDAC. A recent important study in colorectal cancer, revealed associations between CT radiomics and LN metastases [44], thereby providing a reference for this study. We used CT imaging, which is easily accessible as a routine examination method. The 2041 candidate radiomics features extracted from venous phase images were reduced to 15 potential predictors, and the radiomics signature was generated by shrinking the regression coefficients, with the LASSO method. The combined prediction model, including the radiomics signature and clinical characteristics, demonstrated adequate discrimination when compared with the clinical prediction model in the primary cohort (IDI, 0.5046), which was improved in the validation cohort (IDI, 0.3294). This indicated that the radiomics signature was stable and robust for LN metastasis prediction.

The present study had some limitations. Firstly, owing to the nature of a pilot study design, the radiomics analysis was retrospectively applied to single-center data, which lacked external validation. Multicenter data analysis will be incorporated in future studies. Secondly, the radiomics signature of this study was obtained from the venous phase of CECT images, and multimodal parameters were lacking. Finally, we did not stratify the analysis of LN metastasis, although each case had exact pathological results.

## Conclusion

In summary, this pilot study showed that a noninvasive radiomics signature, extracted from CECT images, can be conveniently used to predict preoperative LN metastasis in patients with PDAC.

## Data Availability

The datasets supporting the conclusion of this article available from the corresponding author on reasonable request.

## Abbreviations

AUC: Area under the curve
CA19–9: Cancer antigen-19-9
CEA: Carcinoembryonic antigen
CEST: Contrast-enhanced CT
CT: COMPUTED tomography
IDI: Integrated discrimination improvement
IoU: Intersection-over-union
LASSO: The least absolute shrinkage and selection operator
LN: Lymph node
PDAC: Pancreatic ductal adenocarcinoma
ROC: Receiver operating characteristic
ROI: Region of interest
TBIL: Total bilirubin
